# Epithelial-Myoepithelial Carcinoma of the Breast with Rhabdoid Features

**DOI:** 10.1155/2020/8879035

**Published:** 2020-10-07

**Authors:** Karl Grenier, Gulbeyaz Altinel, Zari Dastani, Atilla Omeroglu

**Affiliations:** ^1^Department of Pathology, McGill University Health Center, 1001 Decarie Blvd, Montreal, Québec, Canada H4A 3J1; ^2^Département de Pathologie, Centre Hospitalier de Lanaudière, 1000 Boulevard Sainte-Anne, Saint-Charles-Borromée, Québec, Canada J6E 6J2

## Abstract

Epithelial-myoepithelial carcinoma of the breast is a rare biphasic tumor composed of intermixed malignant epithelial and myoepithelial components. Myoepithelial cells are known to adopt varied morphologies, including spindle, chondroid, clear cell, and rhabdoid morphologies, and can represent a diagnostic challenge when isolated on biopsy. Rhabdomyosarcoma, phyllodes tumor, metaplastic carcinoma, and myoepithelial carcinoma are primary breast tumors that all have been shown to exhibit rhabdoid features, whether representing true differentiation or morphological mimic. We here report an epithelial-myoepithelial carcinoma of the breast with rhabdoid features in a 76-year-old woman. The rhabdoid-appearing myoepithelial cells are negative for myogenin, consistent with a rhabdoid-like morphology rather than a true rhabdoid differentiation, comparably to previously described myoepithelial carcinoma with rhabdoid features. To our knowledge, this is the first reported case of epithelial-myoepithelial carcinoma of the breast with rhabdoid features and thus adds another entity to the differential diagnosis of breast lesions with rhabdoid features.

## 1. Introduction

Epithelial-myoepithelial carcinomas are biphasic lesions of the breast that include pleomorphic adenoma, adenomyoepithelioma, adenoid cystic carcinoma, and adenomyoepithelioma with carcinoma. The latter normally comprises lesions where the malignant component is derived either from the luminal epithelium or the myoepithelium. When both components are malignant, the preferred term is epithelial-myoepithelial carcinoma (EMC). These, which are derived from a dual population of epithelial and myoepithelial cells, are lesions frequently found in salivary glands but are rare occurrences in breast tissue, with only three dozen or so cases reported in the literature [1–12]. They usually occur in postmenopausal women (mean age 64 years old) but have been found as early as the third decade (39 years old) [12, 13]. It usually presents as a mass that is self-palpated or detected on mammography, with size of 0.7 to 15 cm (mean size 4.5 cm) [12]. While they are usually found arising from a benign adenomyoepithelioma, only the malignant counterpart was found in other cases and thus EMC might also appear *de novo*. Histologically, these lesions are characterized by a biphasic proliferation made of epithelioid, glycogen-rich, or spindled myoepithelial population surrounding epithelial-lined spaces. The architecture of the epithelial component varies from lobulated, tubular, papillary, and mixed with most of the EMC showing multilobulation. The glandular elements can show squamous or apocrine metaplasia and sebaceous differentiation and often show central necrosis or sclerosis. On the other hand, the myoepithelial component can also show spindle, squamous, chondroid, and myxoid morphologies [4, 5, 7, 12, 14]. Otherwise, little is known about genetic rearrangements in these tumors, but two reports have shown mutations in HRAS and PIK3CA [12, 15] and one has shown TP53 mutations in a malignant myoepithelial carcinoma arising from an adenoepithelioma [16]. Here, we show rhabdoid-like morphology of the myoepithelial component, something that was never reported in an epithelial-myoepithelial carcinoma and briefly discuss the implications of this novel entity.

## 2. Case Presentation

**Figure 1:**
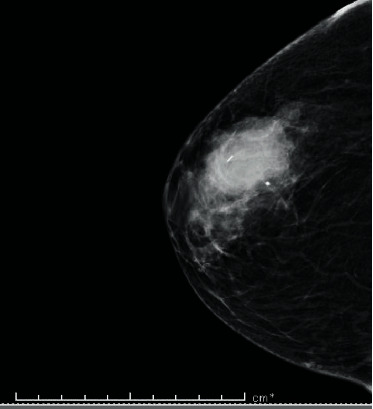
Mammography of the lesion showing a 3-4 cm central mass with no calcifications.

A 76-year-old woman presented with the right breast painful mass at an outside institution. On mammography, the mass measured 3.0 to 4.0 cm and had an ill-defined border (Figure 1). No calcifications were noted. An ultrasound examination revealed a heterogeneous mass with cystic component measuring 3.2 cm, without axillary adenopathy. Patient underwent fine needle aspiration and core needle biopsy, which were suspicious for malignancy but inconclusive for definite diagnosis. Patient underwent total mastectomy with sentinel lymph node biopsy.

## 3. Histopathological Findings

**Figure 2:**
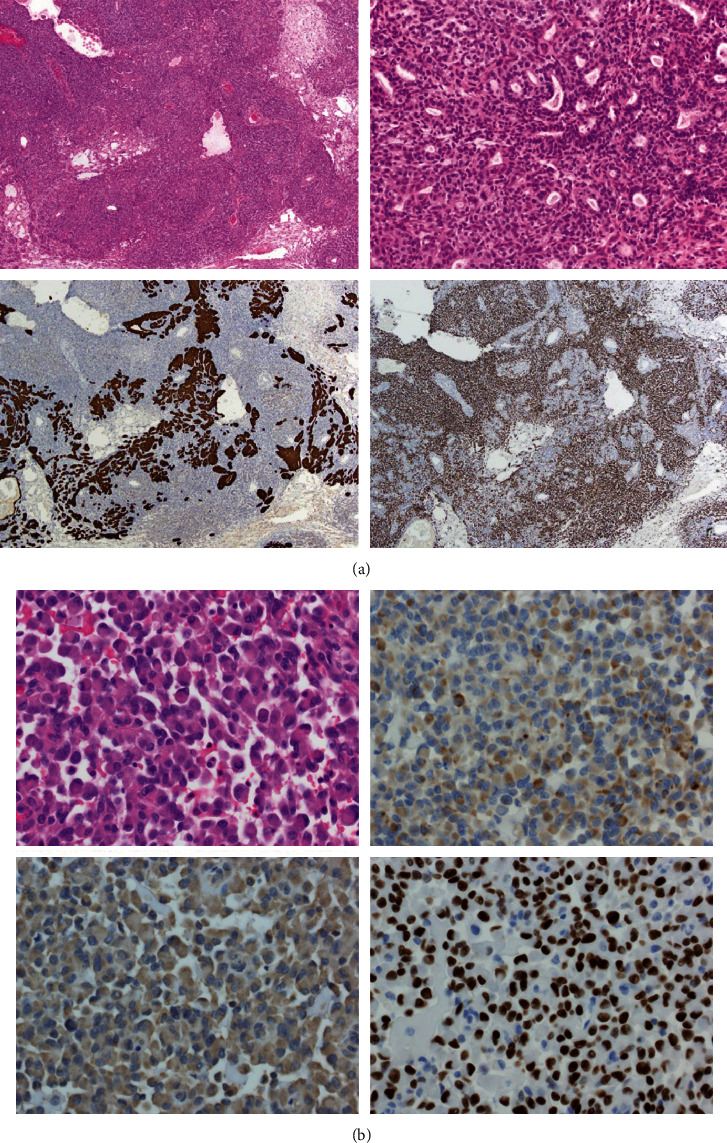
Histopathology of epithelial-myoepithelial carcinoma with rhabdoid features. (a) *Top left*: low-power (40x) representative area with a mixed glandular epithelial-myoepithelial architecture. *Top right*: high-power (200x) representative area with a mixed glandular epithelial-myoepithelial architecture. *Bottom left*: low-power (40x) CK8/18 IHC of the same area. *Bottom right*: low-power (40x) p63 IHC of the same area. (b) *Top left*: high-power (400x) representative rhabdoid area. *Top right*: high-power (400x) myogenin IHC of the same rhabdoid area. *Bottom left*: high-power (400x) CK8/18 IHC of the same rhabdoid area. *Bottom right*: high-power (400x) p63 IHC of the same rhabdoid area.

The mastectomy specimen showed a malignant tumor measuring 4.5 cm with wide clear margins. The tumor was composed of a malignant glandular component (Figure 2(a)) intermixed with more spindle and rhabdoid foci (Figure 2(b)). The second component was strongly positive for P63 and negative for low-molecular-weight cytokeratin (CK8,18) and myogenin, consistent with myoepithelial differentiation (Figure 2(b)). A high mitotic rate of over 10 per 10 high-power fields was identified in the myoepithelial areas, consistent with a high-grade tumor. A diagnosis of epithelial-myoepithelial carcinoma was rendered. There were isolated tumor cells present in 1 out of 2 sentinel lymph nodes.

## 4. Discussion

The tumor reported here showed a biphasic pattern consisting of glandular malignant epithelial structures in a malignant stromal component that showed both spindled epithelioid features and rhabdoid-like morphology. The differential for biphasic breast tumors includes fibroadenoma, pleomorphic adenoma, adenomyoepithelioma, phyllodes tumor, adenoid cystic carcinoma, adenomyoepithelioma with carcinoma, epithelial-myoepithelial carcinoma, and metaplastic carcinoma. With true mesenchymal components, the list shortens to fibroadenoma, phyllodes tumor, and metaplastic carcinoma. However, epithelial-myoepithelial tumors often adopt mesenchymal-like morphologies such as spindled, chondroid, and myxoid stromal background [4, 5, 7, 12, 14]. We now include to this list rhabdoid-like morphology and thus add another caution to avoid misdiagnosing epithelial-myoepithelial carcinoma as metaplastic carcinoma. This appears to constitute a rare event, as rhabdoid-like morphology in a myoepithelial carcinoma of the breast was only reported once, and never in an epithelial-myoepithelial carcinoma [17]. Rhabdoid morphology is best described as round to polygonal cells with eccentric nuclei and abundant cytoplasm with globular paranuclear eosinophilic inclusions that stain positively with myogenin and desmin. Tumors with true rhabdoid differentiation include malignant rhabdoid tumors of childhood (RT) and atypical teratoid rhabdoid tumors (ATRT) and typically exhibit biallelic inactivation mutations of the *SMARCB1* gene [18]. In contrast, tumors with rhabdoid morphology, defined by myogenin or myoD1 expression but no *SMARCB1* alteration, include epithelioid sarcoma, rhabdomyosarcoma, meningiomas, and extra-renal carcinomas [18]. In the breast, tumors with rhabdoid morphology include primary breast rhabdomyosarcoma, phyllodes tumor with heterologous differentiation, and metaplastic carcinoma with mesenchymal differentiation, while tumors with rhabdoid-like morphology only included myoepithelial carcinomas up to now. Interestingly, there are a few other reports of myoepithelial carcinoma of the head and neck and of the vulva with rhabdoid features [19–22]. Some of the myoepithelial carcinoma of the head and neck with rhabdoid features have been found to have loss of nuclear SMARCB1 immunoexpression and EWSR1 rearrangement by *in situ* hybridization, perhaps hinting that malignant myoepithelial cells can partially differentiate towards a rhabdoid fate [21]. In our case, the absence of myogenin and desmin immunoexpression, combined to the p63 immunoexpression positivity, supports a rhabdoid-like morphology rather than a true rhabdoid morphology or differentiation. Given the rarity of this entity, it will be difficult to definitively answer whether rhabdoid features in myoepithelial carcinomas is limited to morphological similarities or if it represents a spectrum towards rhabdoid differentiation. Nevertheless, it will be interesting to continue evaluating these morphological variants histologically and genetically in future studies.
